# Non-Thermal Radio Frequency and Static Magnetic Fields Increase Rate of Hemoglobin Deoxygenation in a Cell-Free Preparation

**DOI:** 10.1371/journal.pone.0061752

**Published:** 2013-04-12

**Authors:** David Muehsam, Parviz Lalezari, Rukmani Lekhraj, Provvidenza Abruzzo, Alessandra Bolotta, Marina Marini, Ferdinando Bersani, Giorgio Aicardi, Arthur Pilla, Diana Casper

**Affiliations:** 1 Department of Neurosurgery, Albert Einstein College of Medicine and Montefiore Medical Center, Bronx, New York, United States of America; 2 Department of Medicine and Pathology, Albert Einstein College of Medicine and Montefiore Medical Center, Bronx, New York, United States of America; 3 Department of Human and General Physiology, University of Bologna, Bologna, Italy; 4 Department of Biomedical Engineering, Columbia University, New York, New York, United States of America; 5 Department of Histology, Embryology and Applied Biology, University of Bologna, Bologna, Italy; 6 Department of Physics, University of Bologna, Bologna, Italy; 7 Interdepartmental Center “L. Galvani”, University of Bologna, Bologna, Italy; 8 Department of Orthopedics, Mount Sinai School of Medicine, New York, New York, United States of America; Sudbury Regional Hospital, Canada

## Abstract

The growing body of clinical and experimental data regarding electromagnetic field (EMF) bioeffects and their therapeutic applications has contributed to a better understanding of the underlying mechanisms of action. This study reports that two EMF modalities currently in clinical use, a pulse-modulated radiofrequency (PRF) signal, and a static magnetic field (SMF), applied independently, increased the rate of deoxygenation of human hemoglobin (Hb) in a cell-free assay. Deoxygenation of Hb was initiated using the reducing agent dithiothreitol (DTT) in an assay that allowed the time for deoxygenation to be controlled (from several min to several hours) by adjusting the relative concentrations of DTT and Hb. The time course of Hb deoxygenation was observed using visible light spectroscopy. Exposure for 10–30 min to either PRF or SMF increased the rate of deoxygenation occurring several min to several hours after the end of EMF exposure. The sensitivity and biochemical simplicity of the assay developed here suggest a new research tool that may help to further the understanding of basic biophysical EMF transduction mechanisms. If the results of this study were to be shown to occur at the cellular and tissue level, EMF-enhanced oxygen availability would be one of the mechanisms by which clinically relevant EMF-mediated enhancement of growth and repair processes could occur.

## Introduction

Identification of some of the mechanisms underlying EMF bioeffects and their clinical applications [1] has contributed to the development and more widespread use of more effective therapeutic signals [2]. It is clear that EMF modulation of nitric oxide signalling plays an important role in EMF therapeutics [2]–[6]. However, there may be other transduction pathways through which EMF signals could modulate tissue repair and growth. For example, several recent studies have reported EMF effects on human Hb in solution and erythrocyte suspensions, including decreases in viscosity [7] and changes in impedance [8], dielectric properties [9], dia- and para-magnetic properties [10], optical absorption [11] and *in vivo* deoxygenation [12]. In addition, radiofrequency mobile telephone signals have been shown to decrease Hb oxygen affinity *in vitro* [13] and changes in infrared absorption have been reported for low-frequency EMF [14]. Also, enhanced oxygen delivery by Hb is under investigation as a therapeutic strategy for the treatment of pathologies such as ischemia from stroke, cardiac disease and diabetic ulcers [15]. Thus, the present study aimed to characterize the effects on the rate of deoxygenation of human hemoglobin (Hb) in an *in vitro* cell-free assay of a pulse-modulated radiofrequency (PRF) signal currently in clinical use for treatment of pain, edema and chronic wounds [1], [2], and, applied independently, of a static magnetic field (SMF), from permanent ceramic magnets constructed for therapeutic applications and reported to reduce pain [1]. These non-thermal EMF modalities were chosen for this study due to their demonstrated efficacy in a variety of therapeutically relevant settings [1]–[6]. The reducing agent dithiothreitol (DTT) was employed for its ability to facilitate control of the time course of Hb deoxygenation by adjusting the ratio of DTT/Hb [16]. This study will show that exposure to PRF and SMF, yielded significant increases in the rate of Hb deoxygenation in the presence of the reducing agent DTT, observable several minutes to several hours after EMF exposure had ended.

## Materials and Methods

### Hemoglobin Preparation

Fresh human blood was obtained with written donor consent and approved for research by the Blood Bank at S.Orsola-Malpighi Hospital, Bologna according to the rules established by Legislative Decree 03-03-2005, article 9, paragraph 3, published in G.U. n. 85, 13.04.2005. Blood samples were also obtained from one author (DM) in accordance with New York State Consolidated Law, Public Health Article 24-A, Section 2442, without written approval of the Institutional Review Board of the Albert Einstein College of Medicine. Blood was drawn in EDTA anticoagulant tubes, centrifuged at 1000 x g for 10 min and plasma removed. The packed erythrocytes were then washed in 0.85% sodium chloride solution and centrifuged at 1000 x g for 10 min, 3 times. The packed, washed erythrocytes were then hemolyzed using distilled water, the resulting solution centrifuged at 1000 x g for 10 min, and the Hb-containing supernatant recovered using a Pasteur pipette. Red cell ghosts were sedimented by additional centrifugation, and the resulting solution containing 2–2.5 mM oxyHb (measured by visible light spectroscopy [17]), was stored in 1.5 mL aliquots at −80°C until used for each experiment. Ten mL reaction mixtures were prepared using 100–120 µM Hb in 50 mM Hepes buffer (pH 7.2, Sigma-Aldrich, USA), and deoxygenation initiated with 20 mM DTT at 22±0.1°C. pH was checked with a digital pH meter (Fisher AB15 BioBasic, USA) and carefully maintained at 7.2 for all hemoglobin samples used in this experiment. Immediately upon addition of DTT, the 10 mL reaction mixture volume was divided into 1 mL aliquots in sealed 1.5 mL microfuge tubes. No attempt was made to alter the gaseous environment within the tubes.

### EMF Exposure

All hemoglobin samples were exposed to the ambient magnetic field, which was measured using a digital Gauss/Tesla meter (model 7010, F.W. Bell, USA) to be 40.5±2 µT, 59 degrees from horizontal (vertical component  = 34.7±2 µT, horizontal component  = 21.0±2 µT). The PRF signal is approved by the US FDA for post-operative pain and edema. The signal consisted of a 27.12 MHz sinusoidal carrier (derived from the carrier frequency reserved and cleared worldwide for short wave diathermy) configured to operate nonthermally through pulse modulation in 4 ms bursts, repeating at 5 Hz and peak magnetic field amplitude of 10±1 µT (Roma3, Ivivi Health Sciences, San Francisco, CA, USA). These pulse modulation parameters were chosen on the bases of theoretical modelling and published reports of bioeffects at the cellular level, and healing at the animal and clinical levels [1]–[3], [18]–[24]. The PRF signal was delivered with a 20 cm circular single-turn antenna (coil) oriented vertically, creating a 10×10×5 cm region of field homogeneity in the central area of the plane of the coil within which a plastic carrier held five upright 1.5 mL microfuge tubes, each containing 1 mL reaction volume. Each Hb sample was contained in a cylindrical volume of 2.5 cm in height and 0.8 cm diameter. For this target size, using Faraday's Law of Induction, the mean peak induced electric field is 3±1 V/m. PRF field parameters were assessed and verified for each experiment using a National Institute of Standards and Testing traceable electrostatically shielded loop probe 1 cm in diameter (model 100A, Beehive Electronics, Sebastopol, CA, USA) connected to a calibrated 100-MHz oscilloscope (model 2012B, Tektronix, Beaverton, OR, USA). The output of the loop probe was calibrated at 27.12 MHz by measurement of output power using a spectrum analyser (model 8567A, Hewlett Packard, New York, NY, USA) and the probe calibration factor for conversion to magnetic field amplitude at 27.12 MHz was certified and given by the manufacturer. SMF exposure was delivered using circular permanent ceramic magnets of 3.8 cm diameter and 1.3 cm thickness, constructed for therapeutic applications (Magnetherapy, West Palm Beach, FL, USA), and composed of compacted and sintered strontium ferrite (SrO-6(Fe_2_O_3_)), encased in plastic. These magnets are axially magnetized to have a single north (N) and a single south (S) pole on each circular face, and manufactured to produce highly uniform field strength across each face. For all experiments, magnets were arranged with circular faces oriented vertically with 1.1 cm gaps between the parallel surfaces. In order to simultaneously expose five 1.5 mL microfuge tubes, two 1.1 cm-wide treatment regions were formed using a central magnet flanked by pairs of magnets on either side. This (NS)(NS)-gap-(NS)-gap-(NS)(NS) configuration produced two cylindrical treatment regions of 1.1 cm width and 3.3 cm in diameter with uniform magnetic field (to±3%), in which the Hb samples were exposed. Within each treatment region the horizontal component of the magnetic field (perpendicular to the magnet surface) was 186±6 mT, a field strength similar to those commonly employed in therapeutic applications [25]. The maximal value of the horizontal and vertical components (both parallel to the magnet surface) was 4.0±0.6 mT. SMF components were measured using a digital magnetometer (Model 450, gaussmeter with MMT-6J02-VG transverse Hall effect probe with 1 mm resolution, Lake Shore Cryotronics, Westerville, OH, USA). For each experiment, five 1.5 mL microfuge tubes containing the hemoglobin preparation were exposed to PRF or SMF for 10–30 min, and 5 tubes were simultaneously exposed to ambient geomagnetic conditions (control) on the laboratory bench, approximately 3 meters away from exposed samples. At this distance contributions from either PRF or SMF to the control condition were undetectable. Temperature variations between exposed and control samples were less than±0.1°C [Fisher AB15 BioBasic, Waltham, MA, USA].

### Spectrophotometric Analysis

After control and EMF exposure, triplicate 300 µL samples were taken from each 1.5 mL microfuge sample tube, pipetted into an open 96-well flat-bottom plate (Fisher Scientific, USA) and the concentrations of oxy, deoxy and metHb were measured spectrophotometrically (SpectraMax 190, Molecular Devices, Sunnyvale, CA, USA). The plate remained in the spectrophotometer at 22°C during the reaction, and the time course of deoxygenation was determined using the method of Benesch et al. [17], which employs a weighted linear combination of optical densities at 560, 576 and 630 nm to determine oxyHb, deoxyHb and metHb concentrations. Stock Hb solutions (in Hepes, pH 7.2) and spectra from Hb/DTT data at t = 0 (i.e. after 30 min EMF/Control exposures) were assayed for oxy, deoxy and metHb at 560, 576 and 630 nm [17]. The mean ratio of deoxy/oxy for Hb stock solutions was 3.9%±0.8%. For the Hb/DTT data at t = 0, the mean deox/oxy was 3.3%±0.6%. The two datasets do not differ significantly (P = 0.44, n = 5). The mean ratio of metHb/oxyHb was 21.8%±3.8% for stock solutions and 25.6%±3.7% for Hb/DTT data at t = 0, indicating no significant differences (P = 0.33, n = 5). Optical densities at 540, 560, 576 and 630 nm were measured immediately after EMF exposure and at successive 1 to 30 min intervals, until maximal deoxygenation was observed. Calibration studies confirmed that the kinetics of deoxygenation could be controlled by adjusting the ratio of DTT/Hb concentrations [16]. Under the conditions stated above, deoxygenation occurred between 140 and 160 min after EMF exposure. Deoxygenation time was reduced by the addition of 5 M of urea to between 60–85 min after EMF exposure. In the absence of DTT, no significant deoxygenation of Hb occurred up to 3 hours (data not shown). Changes in oxyHB concentration with time are summarized in the figures, and maximal differences between deoxy and metHb for EMF exposures vs controls are reported in the text. All figures and data shown are the results of single experiments that have been repeated 2–10 times.

### Statistical Analysis

Results were compared using the Student's t test, or one-way repeated measures ANOVA with Holm-Sidak *post hoc* analysis, and Fieller's method for the variance of ratios, as required (Sigmastat 3.0, Systat, Chicago, IL) and are reported as means±SEM. Significance was accepted at P≤0.05.

## Results

**Figure 1:**
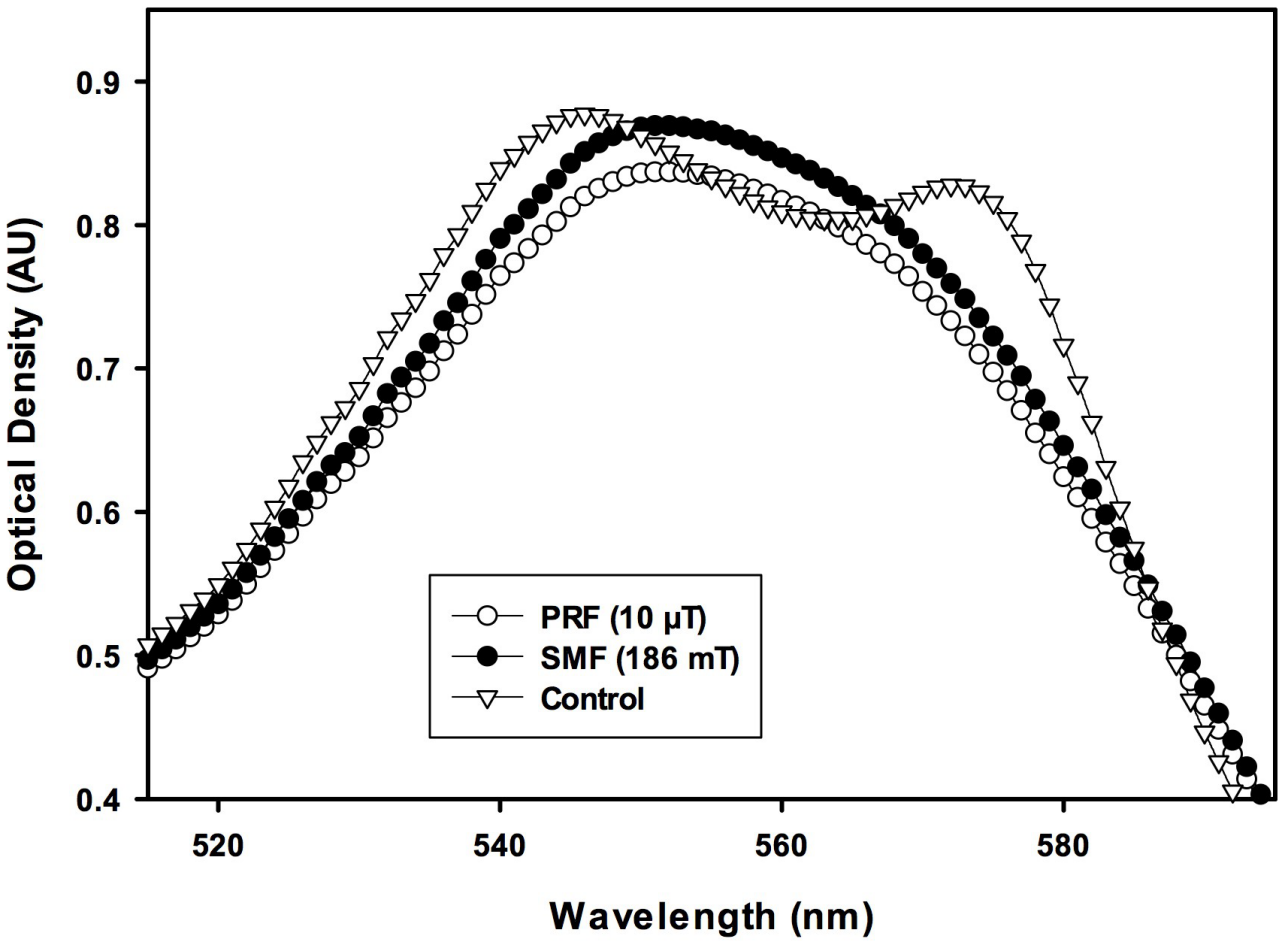
EMF effect on hemoglobin (Hb) visible light spectra. Spectra representative of the effects of pulsed radiofrequency (PRF) signal and 186 mT static magnetic field (SMF) on Hb visible light spectra during bHb)deoxygenation. Data shown are typical of samples drawn from a single tube for each EMF exposure condition, shown here at 150 min after a single 30 min EMF exposure. Deoxygenation of 100 µM Hb was carried out in 50 mM Hepes buffer (pH 7.2) using the reducing agent dithiothreitol (20 mM) at 22°C, and is characterized here by the passage of the spectrum from a two-peaked to one-peaked form. Deoxygenation occured at an earlier time for EMF exposed samples (traces with one peak), as compared to control samples exposed only to the ambient geomagnetic laboratory environment (trace with two peaks). The EMF effect was observable at the time of most rapid deoxygenation.

**Figure 2:**
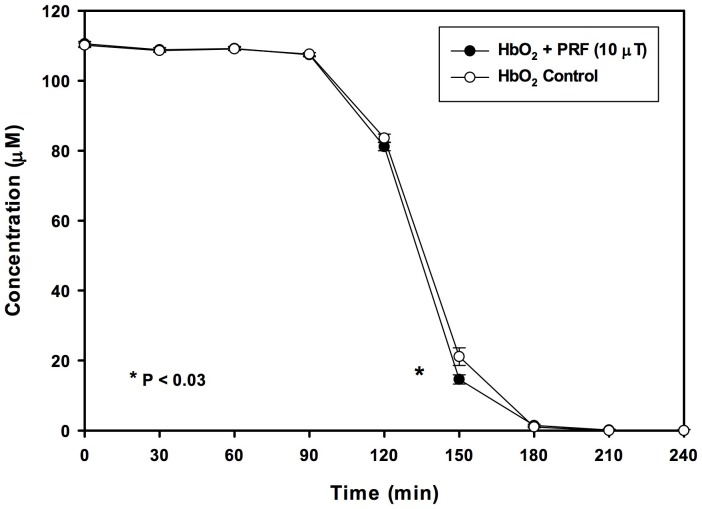
Effect of the pulsed radiofrequency field on time course of hemoglobin deoxygenation. Time course of oxy hemoglobin (HbO_2_) concentration, after a single 30 min pulsed radiofrequency electromagnetic field (PRF) exposure of Hb, under deoxygenating conditions. Concentration was determined by visible light spectroscopy at 560, 576, 630 nm. PRF exposure resulted in a significant (30.8±10.3)% reduction in oxyHb concentration, as compared to controls (14.6±1.3 µM vs. 21.1±2.5 µM, P<0.03, n = 5), suggesting an alteration in Hb solution properties that persisted after PRF signal was removed.

**Figure 3:**
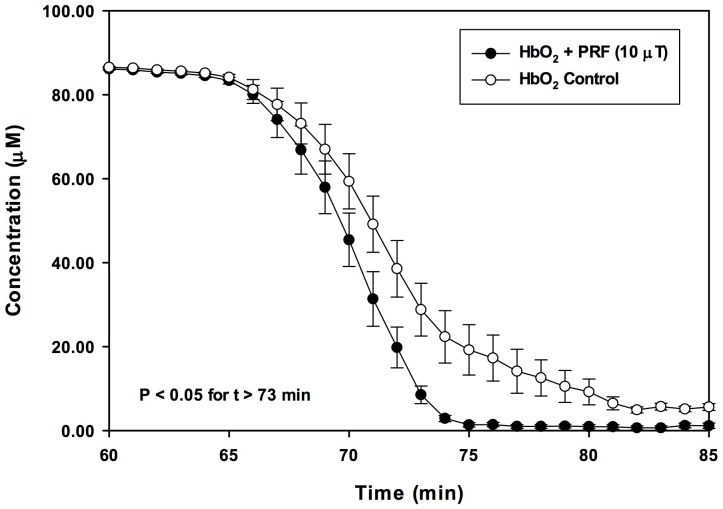
Addition of 5 M urea to reaction mixture. Addition of 5 M urea to the deoxygenation assay reduces the time required for deoxygenation and render the PRF effect more apparent. The maximal difference between PRF-treated and control samples occurred at 73 min, with PRF-treated samples showing a significant (70.4±9.7)% reduction in oxyHb concentration, as compared to controls (8.5±2.1 µM vs. 28.8±6.3 µM, P<0.03, n = 5), in contrast to the 30.8% reduction observed in the absence of urea (cf. Figure 2).

**Figure 4:**
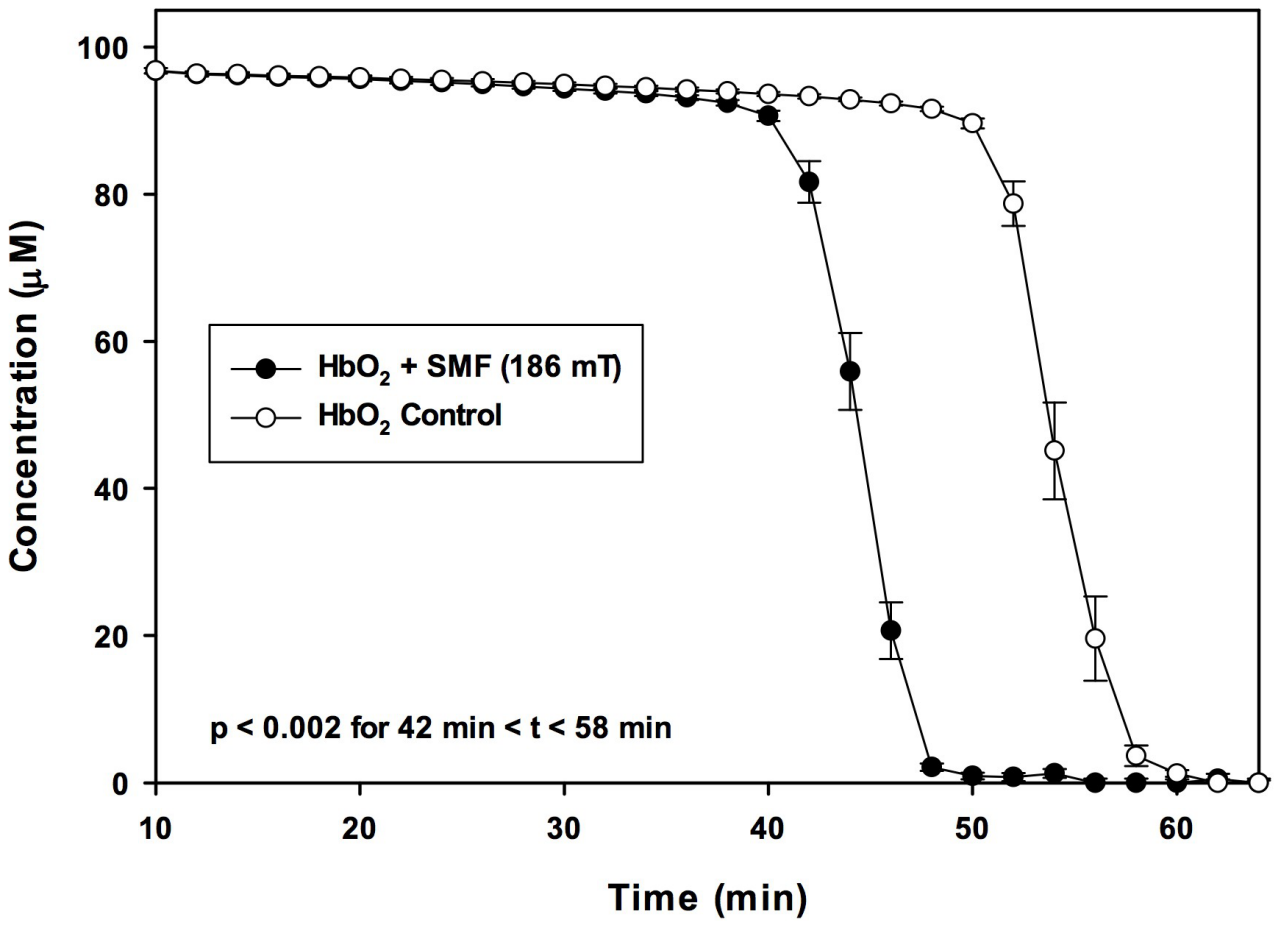
Effect of the static magnetic field on time course of hemoglobin deoxygenation. Hb deoxygenation after 10 min exposure to 186 mT static magnetic field (SMF) in 5M urea. No significant change in oxy/deoxy ratio was visible until the time of rapid deoxygenation, at approximately 40 min. The time of most rapid deoxygenation occurred approximately 10 min earlier for SMF treated samples (P<0.002 for 42 min<t<58 min, n = 5).

**Figure 5:**
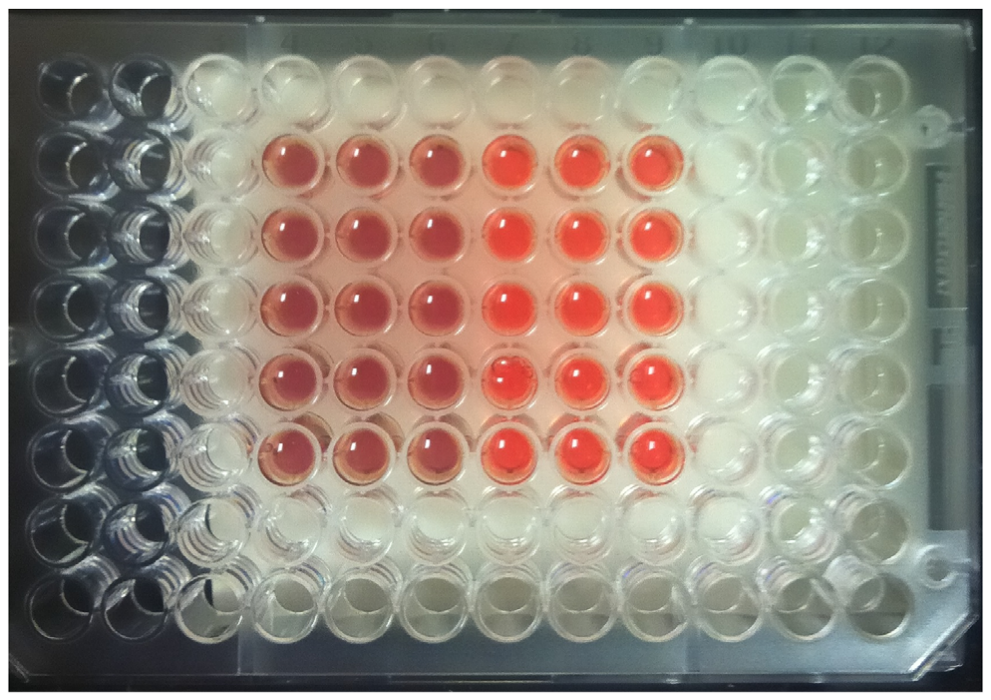
Reaction plate showing that addition of 5M urea to reaction solution renders magnetic field effect more apparent. 96-well spectrophotometer plate shown at 46 min after initiation of reaction of hemoglobin with dithiothreitol (DTT). Samples treated for 10 min with 186 mT static magnetic field (3 left-hand columns in plate) substantially completed (see Figure 4) DTT-induced deoxygenation before untreated samples began to lose O_2_, rendering the magnetic field effect visibly apparent.

Immediately after 30 min of PRF or SMF exposure, no differences in Hb visible light spectra between PRF, SMF and control (ambient magnetic field only) samples were detected. However, changes in visible light spectra were observed in the 540–630 nm region during the time of most rapid deoxygenation, which occurred in this experiment at 140–160 min after EMF exposure. Figure 1 illustrates typical visible light spectra for PRF, SMF and control exposure conditions, drawn from a single exposure tube for each EMF condition. Deoxygenation occurred at an earlier time following PRF and SMF exposures (traces with one peak), as compared to control samples exposed only to the ambient geomagnetic laboratory environment (trace with two peaks). In trials employing PRF exposure (n = 5 tubes for each EMF exposure condition) using 20 mM DTT, 150 min after a single 30 min exposure, PRF-treated samples exhibited a 30.8±10.3% reduction in oxyHb as compared to controls (14.6±1.3 µM vs. 21.1±2.5 µM, P<0.03) (Figure 2). Concomitant, significant increases in deoxy Hb (86.9±1.1 µM vs. 82.8±1.8 µM, P<0.05) and metHb (42.2±1.4 µM vs. 37.1±1.4 µM, P<0.02) also occurred. For the ratio of DTT/oxyHb concentrations employed in these trials (20 mM DTT; 120 µM oxyHb), 150 min corresponded to the time of most rapid deoxygenation. Addition of 5 M urea to the reagent solution decreased the time to required deoxygenate to approximately 65–75 min after reaction initiation. For these conditions, the maximal difference between PRF-treated and control samples occurred at 73 min, with PRF-treated samples showing a 70.5±9.7% reduction in oxyHb as compared to controls (8.5±2.1 µM vs. 28.8±6.3 µM, P<0.03) (Figure 3). Concomitantly, there were significant increases in deoxy Hb (54.4±2.2 µM vs. 40.9±5.2 µM, P<0.04) and metHb (73.4±1.4 µM vs. 61.0±2.3 µM, P<0.02). Under similar conditions (30 mM DTT, 5 M urea, n = 5) SMF treated samples (10–30 min exposure) underwent nearly complete deoxygenation before the untreated (control) samples began to deoxygenate (P<0.002 for 42 min<t<58 min). For these SMF treated samples, the most rapid deoxygenation occurred approximately 10 min earlier than for control samples (Figure 4). Under these conditions, SMF-treated samples substantially completed DTT-induced deoxygenation before untreated samples began to lose O_2_, an effect that was visibly observable in the 96-well spectrophotometer plate, as shown in the photo in Figure 5, taken approximately 46 min after the reaction was initiated. Experiments were also carried out for which: 1) Hb was treated with PRF or SMF for 30 min prior to introduction into the deoxygenation solution (20 mM DTT+5M urea in 50 mM Hepes) and 2) the deoxygenation solution was treated with PRF or SMF for 30 min prior to introduction of Hb. In both cases the difference between PRF- or SMF-treated and control samples was not significant. Also, 10 min and 30 min PRF and SMF treatment durations were compared (in 20 mM DTT+5M urea in 50 mM Hepes), with no significant differences observed in the time course of deoxygenation. In the absence of DTT, no PRF or SMF effects were observed on the Hb oxidation state. The SMF sensitivity of this Hb deoxygenation assay was repeated at the University of Bologna, Italy. Initial experiments were performed in New York with Hb from a single donor, and subsequent trials in Bologna used Hb from 3 different donors, with similar results.

## Discussion and Conclusions

The results from this study indicate that exposure to either PRF or SMF, applied independently, can alter the rate of DTT-induced deoxygenation of human Hb in an *in vitro* cell-free preparation, resulting in more rapid Hb deoxygenation. To the authors' knowledge, this is the first report showing that PRF and SMF produced similar effects in the same biological system, in the same study. The rate of Hb deoxygenation is non-linear in time and is dependent upon the ratio of DTT/HbO_2_. For this assay, the use of DTT as a reducing agent is preferable to other reducing agents, due to the ease with which the time to deoxygenation can be controlled [16]. It is interesting to note that a similar EMF sensitivity, for Hb exposed to a GSM mobile phone signal, was observed using sodium dithionite [13]. This suggests that EMF acted upon a functional aspect of the Hb deoxygenation process itself, and that this effect was not specific to the reducing agent employed in this study. The observation that EMF pre-treatment of Hb alone, or of the deoxygenation solution itself, failed to yield a significant effect suggests that both PRF and SMF exposures acted upon the interaction of Hb with the deoxygenating solution. The addition of 5 M urea reduced the time to deoxygenate and rendered both the PRF and SMF effects more easily observable. Urea creates an extended cloud of waters that only weakly participates in the hydrogen bonding network of bulk water, facilitating the loosening of the protein structure [26] and destabilizing the water-oxyHb structures that act as key allosteric mediators of the Hb T-R transition [27], thus reducing the energy required to deoxygenate. However, it is unclear whether the addition of urea affected the primary PRF or SMF transduction or merely renders the EMF effects more easily observable. No differences were observed between 10 and 30 min SMF and PRF treatment durations. Effects were observable several min to several hours after PRF or SMF exposure was removed, suggesting that these EMF modalities modified protein/solvation structure in a manner that altered the energy required for deoxygenation.

This study does not provide sufficient detail to allow for the elucidation of the mechanisms of action of EMFs on hemoglobin, or to determine if SMF and PRF act upon the same submolecular targets to produce the effects observed here. Also, further studies are required to determine the range of field parameters for which this assay exhibits sensitivity. However, the sensitivity and biochemical simplicity of the assay developed here suggests its usefulness for future studies that may further establish basic biophysical EMF transduction mechanisms. The PRF signal was developed using a theoretical model for the effect of the induced electric field on ion binding kinetics at protein aqueous interfaces [1]–[3], [18]. It should be noted that PRF transduction through the induced electric field is distinct from the transduction pathway involved in SMF effects, for which no electric field is present. This suggests that more than one mechanism may be responsible for the results reported here. Although direct action on ferrous heme [28] or free radical lifetimes [29], [30] may account for the SMF effects observed here, several other models have been proposed for weak, μT-range magnetic field bioeffects [31]. We have previously shown using the Lorentz-Langevin model that μT-range magnetic fields may have an effect on the thermally activated orientation of ionic oscillators and waters bound at the protein surface [32], [33] and that the minimum magnetic field required to directly compete with thermal forces to affect dissociation of a bound ion or ligand from a protein binding site is in the 1–10 mT-range [34]. Thus, the effect of the mT-range magnetic field employed in this study could occur by the direct action of the Lorentz force on charges bound at the protein/water interface. Functionally important hemoprotein molecular motions are slaved to the thermal fluctuations of the bulk solvent [35] and protein hydration plays a fundamental role in the stability of dynamics between Hb T-R conformations [36] so that the PRF and SMF interactions described above, acting at the protein/water interface, may suggest a means by which EMF could modulate protein function.

The deoxygenating conditions employed here, using the reducing agent DTT in an *in vitro* cell-free model, differ substantially from those found *in vivo*. However, EMF-induced changes in the structure and function of Hb and erythrocyte suspensions shown by others [7]–[12], [14] have been reported *in vivo* and in aqueous solution (i.e. without chemical deoxygenating agents), thus demonstrating an EMF sensitivity under physiological conditions and in the absence of reducing agents. Enhanced delivery of oxygen has been shown to reduce inflammation [37] and enhance tissue repair [38] and at least one trial has reported an EMF-induced increase in deoxyHb in an *in vivo* animal model [12]. Allosteric modification of Hb has been suggested as a clinically useful means of enhancing oxygen delivery [39], and is in development for *in vivo* treatment of ischemia from stroke, cardiac disease and diabetic ulcers [15]. Although much further work is required to ascertain the clinical relevance of the results reported here, enhanced oxygen delivery using PRF or SMF may be important non-invasive, non-pharmacologic therapeutic modalities by which clinically relevant EMF-mediated enhancement of growth and repair processes can occur.
