# Correction: Non-Thermal Radio Frequency and Static Magnetic Fields Increase Rate of Hemoglobin Deoxygenation in a Cell-Free Preparation

**DOI:** 10.1371/annotation/21754927-8c35-4076-9054-0b7c9d1ec671

**Published:** 2013-06-21

**Authors:** David Muehsam, Parviz Lalezari, Rukmani Lekhraj, Provvidenza Abruzzo, Alessandra Bolotta, Marina Marini, Ferdinando Bersani, Giorgio Aicardi, Arthur Pilla, Diana Casper

The name of the fourth author was listed incorrectly. The correct name is: Provvidenza M. Abruzzo. The correct citation is: Muehsam D, Lalezari P, Lekhraj R, Abruzzo PM, Bolotta A, et al. (2013) Non-Thermal Radio Frequency and Static Magnetic Fields Increase Rate of Hemoglobin Deoxygenation in a Cell-Free Preparation. PLoS ONE 8(4): e61752. doi:10.1371/journal.pone.0061752. The correct abbreviation in Contributions is: PMA. 

